# Client-perpetrated and husband-perpetrated violence among female sex workers in Andhra Pradesh, India: HIV/STI risk across personal and work contexts

**DOI:** 10.1136/sextrans-2015-052162

**Published:** 2016-02-23

**Authors:** Elizabeth Reed, J T Erausquin, Allison K Groves, Marissa Salazar, Monica Biradavolu, Kim M Blankenship

**Affiliations:** 1 Division of Global Public Health, School of Medicine: La Jolla, University of California, San Diego, CA, USA; 2 Department of Public Health Education, University of North Carolina, Greensboro, Greensboro, NC, USA; 3 Department of Sociology, American University, Washington DC, USA

**Keywords:** CONDOMS, HIV, SEXUAL ASSAULT, SEXUAL BEHAVIOUR

## Abstract

**Objectives:**

This study examines violence experienced in work and personal contexts and relation to HIV risk factors in these contexts among female sex workers (FSW) in Andhra Pradesh, India.

**Methods:**

FSW at least 18 years of age (n=2335) were recruited through three rounds of respondent-driven sampling between 2006 and 2010 for a survey on HIV risk. Using crude and adjusted logistic regression models, any sexual/physical violence (last 6 months) perpetrated by clients and husbands were separately assessed in association with accepting more money for sex without a condom (last 30 days), consistent condom use with clients and husbands (last 30 days), and sexually transmitted infection (STI) symptoms (last 6 months).

**Results:**

The mean age among participants was 32, 22% reported being currently married, and 22% and 21% reported physical/sexual violence by clients and husbands, respectively. In adjusted logistic regression models, FSW who experienced client violence were more likely to report accepting more money for unprotected sex trades (adjusted OR (AOR)=1.7; 95% CI 1.4 to 2.2), less likely to report consistent condom use with clients (AOR=0.6; 95% CI 0.5 to 0.7) and more likely to report STI symptoms (AOR=3.5; 95% CI 2.6 to 4.6). Women who reported husband violence were more likely to report accepting more money for unprotected sex trades (AOR=2.1; 95% CI 1.2 to 3.7), less likely to report consistent condom use with clients (AOR=0.5; 95% CI 0.3 to 0.8) and more likely to report STI symptoms (AOR=2.6; 95% CI 1.6 to 4.1).

**Conclusions:**

Among FSW, experiences of violence in *work* and *personal* contexts are associated with sexual HIV risk behaviours with clients as well as STI symptoms.

## Introduction

In India, 2.4 million people are estimated to be living with HIV.^1^ ^2^ While HIV prevalence is 0.25% among women in India, the prevalence among female sex workers (FSW) has been estimated at 5%,^3^ ^4^ and as high as 55% in select regions.^5^ ^6^ The southern Indian state of Andhra Pradesh is among the Indian states with the highest rates of HIV; FSW are among the groups largely affected in this region.^7^

Increasingly, research among FSW in India has focused on factors that challenge women's ability to engage in sexual behaviours protective of HIV. One such factor is violence. FSW in India and globally experience various forms of violence (eg, perpetrated by police, clients and non-paying relationship partners).^2^ ^8–12^ Sexual violence has been documented to increase HIV risk because perpetrators are not likely to use condoms,^13^ ^14^ and also because such violence is likely to result in lacerations and vaginal trauma.^2^ ^15^ ^16^ Additionally, previous studies have reported clients’ use of violence and threats of violence as a way to demand having sex without a condom.^17^ ^18^

The majority of this previous work among FSW has focused on physical and sexual violence from clients and/or police and the relation of such violence to condom use during sex trades. Despite high levels of violence perpetrated by non-commercial relationship partners, including husbands,^19^ ^20^ among FSW, the effects of such violence have primarily been discussed in terms of women's HIV risk behaviours with these same non-commercial partners/husbands—not in relation to women's condom use with *clients*. It has been very well documented in studies among other populations that women who have histories of partner violence or sexual abuse are less likely to use condoms with subsequent relationship partners, potentially as a result of lower perceptions of condom negotiating power in sexual relations.^21–23^ Such findings could have implications for FSW, for example, regarding whether experiencing violence in one's personal life may affect condom use in the context of sex trades. Furthermore, an increasing number of studies have indicated that vulnerabilities within the personal lives of FSW (eg, high levels of responsibility in the caretaking of children, housing instability, high levels of mobility, economic debt and financial dependence)^24–28^ may increase the urgency of women's work, thereby increasing women's sexual HIV risk behaviours and likelihood of sexually transmitted diseases (STIs). Given the high proportions of FSW experiencing violence from husbands or non-paying intimate partners in other settings,^19^ ^20^ it is important to better understand both women's experiences of partner violence and its potential impact on HIV risk behaviours with both relationship partners and clients in Andhra Pradesh. Thus, more work is needed to understand how experiences of violence across *personal* and *work-*related contexts of women's lives may each contribute to HIV risk among FSW.

The primary objective of the current study is to examine violence experienced in both work (eg, by clients) and personal (eg, by husbands) contexts and relation to HIV risk factors in these contexts, including whether violence experienced in one context (eg, personal) is associated with HIV risk factors in another context (eg, work) among FSW in Andhra Pradesh, India.

## Methods

### Procedure and sample

Quantitative data are from the three rounds of cross-sectional survey data collected among FSW between 2006 and 2010. Data collection was part of the Project Parivartan, which aims to evaluate the impact of a community mobilisation intervention on HIV risk among FSW in Rajahmundry, an area in the East Godavari District of Andhra Pradesh, India. Eligible participants were at least 18 years of age, female and reported having sex in exchange for money in the year prior to the survey. For each of three rounds of data collection (2006, 2007 and 2009–2010), participants were recruited via respondent-driven sampling (RDS). Specific RDS recruitment procedures are described in greater detail in our previous work.^27^ The RDS recruitment process continued until the predetermined sample sizes were met. Among FSW who received coupons and came to take the survey, few were ineligible (less than 5%) in each round of data collection. Furthermore, we had a high number of recruitment waves for each round of data collection, indicating that the samples from each round were adequate in representing the underlying population of FSW in this region.^29^ ^30^ We describe the sampling strategy and survey development in more detail in our previous work.^24–28^

Trained female Telugu-speaking research assistants obtained informed consent and administered the 90–120 min surveys in Telugu in a private Project Parivartan field office. Upon completion of surveys, participants were provided 200 rupees (approximately US$4.70) as compensation for their time and transportation costs, as well as an additional incentive for each coupon that resulted in new study participants. Participants were offered referrals and accompaniment to relevant local support services as needed. This research was approved by the Human Research Protections Programme at the University of California (protocol #131057), as well as the Institutional Review Boards at American University, Duke University, Yale University, and the VHS-YRG Care Medical Centre Institutional Review Board in Chennai, India. A total of 812 FSW in round I, 673 in round II and 850 in round III gave informed consent and completed the survey. No participants were missing data on the sexual risk outcome variables and, therefore, all were included in the current analysis.

### Quantitative measures

#### Demographic variables

*Age* was measured as a continuous variable. *Marital status* (whether report being currently married), *children* (any, under age 5, between the ages of 5 and 18) and the *venue* in which they worked (brothel, street, lodge or hotel, home, highway or other) were also assessed.

#### Violence

*Physical violence* (last 6 months) was measured by combining two items; participants were grouped as experiencing physical violence if they responded positively to either (1) experiencing being beaten (eg, hit, slapped, pushed, kicked, punched, choked or burned) or (2) threatened with a knife, gun or other weapon, or had a weapon used against them. *Sexual Violence* (last 6 months) was measured by asking participants whether anyone forced them to have vaginal, anal or oral sex against their will in the last 6 months (yes/no). Women were also asked to identify the perpetrator of each type of violence (client, husband, relationship partner, police, stranger).

#### Sources of HIV risk

*Accepting more money for sex without a condom* was measured by asking participants whether this happened in the last 30 days (yes/no). *Consistent condom use* was measured by asking participants how often they used condoms with clients in the last 30 days; participants who reported ‘always’ using condoms with clients were categorised as consistent condom users. Participants were asked the same set of questions regarding their condom use with husbands. Participants were grouped as experiencing *STI symptoms* in the 6 months prior to the survey if they reported experiencing any of the following symptoms: lower abdominal pain not related to diarrhoea or menses, foul smelling vaginal discharge, burning while urinating, genital ulcers/sores, swelling in groin area, itching.

### Data analyses

Frequencies were assessed for sample characteristics and reports of physical and sexual violence by clients and husbands. Given the similar patterns in the data in relation to the outcome variables, experiences of any physical and/or sexual violence were combined into one variable for each perpetrator type. Due to small numbers reporting non-marital relationship violence (6% reporting physical violence and 1% reporting sexual violence perpetration) as well as police and stranger violence (all under 5%), analyses were restricted to violence perpetrated by husbands and clients. Logistic regression models were used to assess any physical and/or sexual violence in relation to accepting more money for unprotected sex trades, consistent condom use with clients, consistent condom use with husbands and STI symptoms. Separate models were constructed for assessing the influence of violence perpetrated by clients and violence perpetrated by husbands on these HIV risk-related variables. Sample characteristics associated with physical or sexual violence in bivariate analyses at p<0.05 were included in adjusted models. Adjustments were also made to consider correlations between these different types of violence perpetrated (eg, models assessing client-perpetrated violence were adjusted for husband-perpetrated violence). ORs are presented with associated 95% CIs, and significance of individual variables was evaluated using Wald χ^2^ tests. All analyses were conducted using SAS V.9.1 (SAS Institute, Cary, North Carolina, USA).

## Results

### Sample characteristics

The mean age of women in the sample was 32 (SD=8.2), and 78% were under the age of 40. Most women (86%) had less than a high school education and 22% reported being currently married. The majority of women (90%) report having at least one child. Women work in various types of venues, including at home (25%), on the highway (19%), street (11%), lodge or hotel (7%) and brothels (4%) (table 1). A detailed description of sample characteristics by study wave has been provided in our previous work.^30^

**Table 1:** Sample characteristics: total and by reported client and husband violence (N=2336)

Variable	Total sample (N=2335) % (n)	Client violence n=520 (80.4%) % (n)	No client violence n=1816 (19.6%) % (n)	Husband violence n=110 (80.4%) % (n)	No husband violence n=405 (80.4%) % (n)
Age					
18–24 years	15.8 (370)	18.9 (98)	15.0 (272)	19.1 (21)	13.1 (53)
25–29 years	21.5 (501)	21.9 (114)	21.3 (387)	28.2 (31)	20.7 (84)
30–34 years	18.4 (429)	19.8 (103)	18.0 (326)	20.2 (22)	19.5 (79)
35–39 years	22.1 (515)	22.1 (115)	22.0 (400)	24.6 (27)	23.7 (96)
40 and older	22.3 (521)	17.3 (90)	23.7 (431)	8.2 (9)	23.0 (93)
		χ^2^=12.1; p=0.01		χ^2^=13.9; p=0.0076	
Education					
Less than high school	86.3 (2006)	84.6 (438)	86.8 (1568)	89.8 (97)	80.2 (323)
High school	13.0 (303)	15.1 (78)	12.5 (225)	10.2 (11)	19.4 (78)
College or technical training	0.7 (15)	13.3 (2)	0.7 (13)	0 (0)	0.5 (2)
		χ^2^=3.0; p=0.22		χ^2^=5.6; p=0.06	
Married					
Yes	22.0 (515)	19.1 (99)	22.9 (416)	–	–
No	78.0 (1821)	80.1 (421)	77.1 (1400)	–	–
		χ^2^=3.5; p=0.06			
Children					
Yes	89.9 (2100)	90.0 (468)	89.9 (1632)	94.6 (104)	91.4 (370)
No	10.1 (236)	10.0 (52)	10.1 (184)	5.5 (6)	8.6 (35)
		χ^2^=0.07; p=0.93		χ^2^=1.2; p=0.27	
Venue of work†					
Home-based	25.1 (586)	25.7 (466)	23.1 (120)	17.3 (19)	20.3 (82)
Highway-based	19.1 (445)	26.0 (135)	17.1 (310)	11.8 (13)	12.1 (49)
Street-based	11.0 (256)	9.4 (49)	11.4 (207)	8.2 (9)	8.4 (34)
Brothel-based	6.7 (156)	9.0 (47)	6.0 (109)	14.6 (16)	6.7 (27)
Lodge	3.9 (90)	5.8 (30)	3.3 (60)	3.6 (4)	3.0 (12)
Agricultural field	15.8 (368)	13.7 (71)	16.4 (207)	17.3 (19)	25.1 (101)
Other	18.9 (428)	12.7 (66)	20.0 (362)	27.3 (30)	24.3 (98)
		χ^2^=44.7; p<0.001		χ^2^=9.4; p=0.15	

Numbers may not add up to 100% due to missing data.

†Numbers may exceed 100%; categories are not mutually exclusive.

Women who reported experiencing client violence or husband violence were more likely to be in younger age categories compared with women not experiencing these forms of violence (p=0.01 and p=0.0076, respectively) (table 1). Women who reported violence from husbands were more likely to report less than a high school education compared with those not reporting such violence; however, this was of borderline significance (p=0.06). Women who reported violence from clients were more likely to be married compared with those not reporting such violence; however, this was of borderline significance (p=0.06). Different types of venues were also associated with greater reports of client violence as well (particularly highway, brothel and hotel/lodge-based venues) (p<0.0001).

### Physical and sexual violence by perpetrator type and HIV risk factors

Overall, 29% reported physical violence (any perpetrator) and 16% reported sexual violence (any perpetrator) (not reported in table). Violence was most commonly reported to be perpetrated by clients and husbands; 22% and 21% reported physical or sexual violence by clients and husbands, respectively. Physical violence perpetrated by husbands was the most common form of violence reported (21%). Physical violence perpetrated by clients (13%) was also common in this sample (table 2). Sexual violence perpetrated by husbands was reported to be less common (1%); however, 13% reported sexual violence perpetrated by clients.

**Table 2:** Violence type and perpetrator (previous 6 months) (n=2336)

Perpetrator	Physical violence % (n)	Sexual violence % (n)	Any physical or sexual violence % (n)
Client			
** **Yes	13.1 (307)	13.1 (307)	22.3 (520)
No	86.9 (2029)	86.9 (2029)	77.7 (1816)
Husband (n=515)*			
Yes	20.8 (107)	1.0 (5)	21.4 (110)
No	79.2 (408)	99.0 (510)	78.6 (405)

*Among those who report having husbands.

Accepting more money for sex with no condom used was reported by 16%. Consistent condom use (last 30 days) among clients was reported by 56%; however, consistent condom use among husbands was reported by 11%. Almost one half (48%) reported having an STI symptom in the previous 6 months (table 3).

**Table 3:** Violence from clients and husbands: relation to sexual risk factors for HIV (n=2336)

	More money for sex with no condom, last 30 days 15.6% (n=365)	Consistent condom use with clients, last 30 days 56.6% (n=1323)	Consistent condom use with husbands/partners, last 30 days 10.7% (n=55)*	STI symptom, last 6 months 47.6% (n=1113)
Violence by perpetrator type (independent variable in all models)	OR (95% CI)	OR (95% CI)	OR (95% CI)	OR (95% CI)
Client-perpetrated violence				
Yes				
Crude	1.7 (1.4 to 2.3)†	0.6 (0.5 to 0.8)†	1.0 (0.5 to 2.0)	2.9 (2.3 to 3.6)†
Adjusted‡	1.7 (1.4 to 2.2)†	0.6 (0.5 to 0.7)†	0.9 (0.4 to 2.0)	2.9 (2.3 to 3.6)†
No	1.0 (referent)	1.0 (referent)	1.0 (referent)	1.0 (referent)
Husband-perpetrated violence§				
Yes				
Crude	1.9 (1.1 to 3.1)¶	0.5 (0.3 to 0.8)¶	0.7 (0.3 to 1.6)	2.4 (1.5 to 3.8)**
Adjusted‡	2.1 (1.2 to 3.7)¶	0.5 (0.3 to 0.8)¶	0.8 (0.3 to 1.8)	2.6 (1.6 to 4.1)†
No	1.0 (referent)	1.0 (referent)	1.0 (referent)	1.0 (referent)

*Analyses restricted to those reporting having sex with husbands in the last 30 days (n=325).

‡Adjusted logistic regression models adjusted for age, venue, study round and other violence (eg, models assessing client-perpetrated violence are adjusted for husband-perpetrated violence).

§Analyses restricted to those reporting husbands (n=515).

¶p<0.05, Wald.

**p<0.001.

†p<0.0001.

### Client-perpetrated and husband-perpetrated violence and relation to risk factors for HIV: findings from crude and adjusted logistic regression models

In logistic regression models adjusted for demographics and other forms of violence perpetration, women who reported physical and sexual violence perpetrated by clients were more likely to report accepting more money for unprotected sex trades (last 30 days) (adjusted OR (AOR)=1.7; 95% CI 1.4 to 2.2), less likely to report consistent condom use with clients (last 30 days) (AOR=0.6; 95% CI 0.5 to 0.7) and more likely to report STI symptoms (last 6 months) (AOR=3.5; 95% CI 2.6 to 4.6). Women who reported physical and sexual violence perpetrated by husbands were also more likely to report accepting more money from clients for unprotected sex trades (last 30 days) (AOR=2.1; 95% CI 1.2 to 3.7), less likely to report consistent condom use with clients (last 30 days) (AOR=0.5; 95% CI 0.3 to 0.8) and more likely to report STI symptoms (last 6 months) (AOR=2.6; 95% CI 1.6 to 4.1). Crude logistic regression models produced similar findings. There were no significant findings between client-perpetrated or husband-perpetrated violence and consistent condom use with husbands (table 3).[Supplementary-material SM1]

10.1136/sextrans-2015-052162.supp1 Supplementary references

## Discussion

Our findings highlight the extent and varying forms of violence within the lives of FSW as well as the impact of this violence on HIV risk. Most noteworthy, this is the first research study among FSW, to our knowledge, to indicate that violence perpetrated by husbands is associated with reduced condom use with clients, including accepting more money for sex without a condom.

Present study findings build on a number of studies documenting the link between experiences of violence among FSW and increased STI and sexual risk behaviours for HIV.^W1–W12^ This previous work has primarily focused on the association between violence perpetrated by non-commercial partners or clients and unprotected sex within these same partner types.^W1–W3^ Not reported in this previous work, our findings specifically document that violence perpetrated by husbands appears to be associated with women's non-condom use with clients. Findings of the present study are supported by previous work indicating that a number of aspects related to women's personal lives (eg, economic vulnerability, high levels of responsibility in the caretaking of children, housing instability, mobility) appear to increase the urgency of women's work, thereby increasing women's sexual HIV risk behaviours and likelihood of having an STI.^24–28^

Regarding possible mechanisms through which husband-perpetrated violence may produce reduced condom use with clients, previous studies have documented that some non-commercial male partners of FSW (including husbands) may become involved with women's sex trades as a broker, and have forced women to have sex with clients without using condoms for greater financial returns.^W6–W14^ Violence perpetrated by husbands may also reduce women's perceptions regarding their condom negotiating power in the context of sex trades with clients, or affect the urgency of women's work in other ways. Furthermore, reduced perception of condom and sexual negotiating power has been well documented in previous works among other populations of women who have histories of partner violence or sexual abuse.^21–23^

The link between client-perpetrated and husband-perpetrated violence and accepting more money for sex trades without a condom provides some evidence of the possible contribution of economic factors in explaining the association between victimisation and increased HIV risk behaviours (eg, non-condom use during sex trades with clients to make more money). Notably, we conducted exploratory analyses to assess whether women's reports of economic debt mediated the relation between violence and HIV risk factors, but we did not find a significant effect. However, other types of economic vulnerability may be more important than debt in contributing to the association between violence and HIV risk among FSW. Future studies are needed that consider other types of economic vulnerability to better understand whether experiences of client-perpetrated and/or husband-perpetrated violence create increased economic vulnerability among FSW, which in turn limit condom negotiating power with clients.

Findings of the present study are also consistent with the previous literature documenting the low rates of condom use with husbands of FSW. Condom use has been shown across multiple studies to be infrequent among non-commercial partners, often as a way to establish trust and intimacy.^W1 W7–W9^ We did not find an association between violence perpetrated by husbands and condom use with husbands; this may likely be related to the high rates of inconsistent condom use with husbands (89%). While we found an association between experiencing violence from husbands and STI symptoms, there was not a significant association between husband-perpetrated violence and non-condom use with husbands. Thus, our findings suggest that increased STIs related to violence from husbands might be partially explained by increased non-condom use with clients among women experiencing violence from husbands. While more work is needed to better understand the mechanisms explaining these findings, this finding is new to the literature.

Consistent with previous research, the current study found that FSW experienced high rates of both physical and sexual violence, from both commercial and non-commercial partners^12^ ^W14^. However, the prevalence of physical and sexual violence across studies varies considerably, often due to varying measures and whether the definition was limited to specific perpetrator types or based on different time periods measured (recent vs ever).^W2 W5^ Furthermore, the under-reporting of violence due to high levels of associated stigma^W13–W15^ may vary by population, and be of particular relevance in more rural contexts such as Rajahmundry, Andhra Pradesh where the current study was conducted.

The findings of this study must be considered with recognition of several limitations. The cross-sectional design does not establish the temporality of these associations; however, our findings build on previous work linking victimisation to subsequent non-condom use, including findings from longitudinal studies. Additionally, stigma can often result in under-reporting of sensitive issues or socially undesirable behaviours, such as violence and non-condom use.^W13–W15^ Such under-reporting would decrease power to detect an association between violence and non-condom use. The current study found multiple strong links among these factors. Because violence perpetrated by non-marital male partners was reported in low proportions, we did not have sufficient sample size to examine whether violence perpetrated by these other relationship partners may also have an effect on sexual risk behaviours for HIV. Future work is needed to consider violence perpetrated by these other types of non-commercial partners. Additionally, our assessment of self-reported STI symptoms would be improved in future studies with the use of biological markers detecting STI infection. Regarding study recruitment, while RDS cannot guarantee a sample truly representative of the underlying population, previous studies have found RDS to be an effective method for sampling ‘hard to reach’ populations, including FSW.^3–6^ Furthermore, the current study findings are most applicable to FSW in Rajahmundry, Andhra Pradesh and may not be generalisable to populations of FSW elsewhere.

These limitations notwithstanding, we document the high prevalence of violence among FSW in this South Indian context, as well as the implications of such violence on women's risk for HIV. Present study findings highlight that experiences of violence in *work* and *personal* contexts are associated with sexual HIV risk behaviours with clients as well as STI symptoms. Thus, efforts are needed to address multiple forms of violence and perpetrators of violence in order to maximise HIV/STI prevention benefits. Notably, violence *in itself* (independent of any associations it may have with HIV/AIDS or other health outcomes) is a critical health and human rights issue affecting FSW across multiple domains in their lives.

**Key messages**

- Violence is a major contributing factor to HIV risk among female sex workers (FSW).
- Violence experienced from husbands is associated with HIV risk behaviours in the exchange of sex at work.
- Contextual-level HIV prevention strategies are needed to address multiple forms of violence and perpetrators of violence.
