# Supplementary material for: Client-perpetrated and husband-perpetrated violence among female sex workers in Andhra Pradesh, India: HIV/STI risk across personal and work contexts
Source: Sex Transm Infect. 2016 Feb 23;92(6):424–9. doi: 10.1136/sextrans-2015-052162 (PMC4992639; doi:10.1136/sextrans-2015-052162)
Supplement: Supplementary references [file sextrans-2015-052162supp_references.pdf]

W1. Mooney A, Kidanu A, Bradley H. et al. Work-related violence and inconsistent condom use with non-paying partners among female sex workers in Adama City, Ethiopia. Bmc Public Health. 2013;13:771.

W2. Deering KN, Bhattacharjee P, Mohan HL, et al. Violence and HIV risk among female sex workers in Southern India. Sex Transm Dis. 2013;40(2):168-74.

W3. Lang DL, Salazar LF, DiClemente RJ, et al. Gender based violence as a risk factor for HIV-associated risk behaviors among female sex workers in Armenia. AIDS Behav. 2013;17(2):551-8.

W4. Karandikar S, Próspero M. From client to pimp: Male violence against female sex workers. Journal of Interpersonal Violence. 2010;25(2):257-273.

W5. Decker MR, Pearson E, Illangasekare SL et al. Violence against women in sex work and HIV risk implications differ qualitatively by perpetrator. BMC Public Health. 2013;13:876.

W6. Bhattacharjee P, Isac S, Raghavendra T, et al. *Role of lovers/ boyfriends in HIV transmission among female sex workers.* Bengaluru, India: Karnataka Health Promotion Trust; 2009.

W7. Panchanadeswaran S, Johnson S, Sivaram S, et al. Intimate partner violence is as important as client violence in increasing street-based female sex workers' vulnerability to HIV in India. The International Journal on Drug Policy. 2008;19(2):106-112.

W8. Murray L, Moreno L, Rosario S, et al. The role of relationship intimacy in consistent condom use among female sex workers and their regular paying partners in the Dominican Republic. AIDS and Behavior. 2007;11(3):463-470.

W9. Voeten, HA, Egesah OB, Varkevisser CM, et al.. Female sex workers and unsafe sex in urban and rural Nyanza, Kenya: regular partners may contribute more to HIV transmission than clients. Trop Med Int Health. 2007;12(2):174-82.

W10. George A, Sabarwal S. Sex trafficking, physical and sexual violence, and HIV risk among young female sex workers in Andhra Pradesh, India. Int J Gynaecol Obstet. 2013;120(2):119-23.

W11. Odinokova V, Rusakova M, Urada LA et al. Police sexual coercion and its association with risky sex work and substance use behaviors among female sex workers in St. Petersburg and Orenburg, Russia. Int J Drug Policy. 2013;25(1):96-104.

W12. Elmore-Meegan M, Conroy RM, Agala CB. Sex workers in Kenya, numbers of clients and associated risks: an exploratory survey. Reprod Health Matters. 2004;12(23):50-7.

W13. Hanck, SE, Blankenship, KM, Irwin, KS, et al. Assessment of self-reported sexual behavior and condom use among female sex workers in India using a polling box approach: a preliminary report. Sex Transm Dis.2008;35(5):489-94.

W14. Botta, RA, Pingree S. Interpersonal communication and rape: women acknowledge their assaults. J Health Commun. 1997;2(3):197-212.

W15. Fisher BS, Daigle LE, Cullen FT, et al. Acknowledging sexual victimization as a rape: Results from a national-level study. Justice Quarterly. 2003;20:535-574.
